# Increased prevalence of hepatitis C virus subtype 6a in China: a comparison between 2004–2007 and 2008–2011

**DOI:** 10.1007/s00705-014-2185-1

**Published:** 2014-08-02

**Authors:** Xia Rong, Ru Xu, Huaping Xiong, Min Wang, Ke Huang, Qiuyu Chen, Chengyao Li, Qiao Liao, Jieting Huang, Wenjie Xia, Guangping Luo, Xin Ye, Ming Zhang, Yongshui Fu

**Affiliations:** 1 Guangzhou Blood Center, 31 Lu Yuan Road, Guangzhou, 510095 Guangdong China; 2 Department of Transfusion Medicine, School of Biotechnology, Southern Medical University, Guangzhou, Guangdong China; 3 Department of Epidemiology and Biostatistics, Faculty of Infectious Diseases, University of Georgia, Rm226 Miller Hall Building, Health Sciences Campus, Athens, GA 30622 USA

## Abstract

Different hepatitis C virus (HCV) genotypes exhibit differences in disease pathogenesis and progression, as well as disease outcomes and response to therapy. Tracking the change of HCV genotypes in various epidemiological settings is critical for both disease surveillance and the development of improved antiviral treatment. Here, we tracked the changes in the prevalence of the HCV genotypes in China between 2004-2007 and 2008-2011. HCV-RNA-positive sera were collected from volunteer blood donors during the period 2008-2011. The genotypes were determined by phylogenic analysis using the NS5B and E1 sequences. Geographical and demographic distribution patterns related to the HCV genotypes obtained in 2008-2011 were compared with our previous study, which recorded data in the period 2004-2007. Pearson chi-square test and t-test were used to statistically analyze the results. In 2008-2011, HCV subtypes 1b and 6a were detected in 43.8 % (184/420) and 34.3 % (144/420), respectively. The male/female ratio was found to be higher for HCV genotype 6 than for genotypes 1 and 2. When compared with the period of 2004-2007, although no significant difference was found in gender or age for genotypes 1, 2, 3 and 6, the subtype 6a frequency was significantly increased from 11 % to 26.5 % in the blood donors from outside of Guangdong Province in 2008-2011. A pattern of increase in HCV subtype 6a was found in blood donors outside of Guangdong Province, indicating that HCV subtype 6a has rapidly spread from Guangdong to other regions of China over the past 10 years.

**Electronic supplementary material:**

The online version of this article (doi:10.1007/s00705-014-2185-1) contains supplementary material, which is available to authorized users.

## Introduction

Hepatitis C virus (HCV) is one of the major causative agents of chronic liver disease [1]. HCV is classified into 7 genotypes and 67 subtypes [2] based on genetic diversity. Subtype 1b is prevalent worldwide [3], while the other genotypes are typically confined to regional epidemics. In brief, subtype 1a is most commonly seen in the USA [4]; subtype 2a and 2b are predominant in North America, Europe and Japan [5–7]; HCV-3 (predominantly 3a) is the most prevalent genotype in India and Pakistan [8, 9]; genotype 4 is often found in the Middle East and Africa [3]; subtype 5a accounts for at least 50 % of the infections in South Africa [10] and genotype 6 (HCV-6) is frequently seen in southern China and Southeast Asia [11–13]. Furthermore, the genotype reportedly confers a different individual response to the commonly used combination therapy interferon (IFN) + ribavirin (RBV). For instance, genotype 1 was shown to be refractory compared with the responses of genotypes 2 and 3 [14, 15]. With the recent availability of two newly approved direct-acting antiviral (DAA) drugs (telaprevir and boceprevir), patients infected with genotype 1 have exhibited an increased sustained virologic response (SVR) rate in short courses of treatment [16]. At present, there is no standard therapeutic regimen for patients infected with genotype 6.

HCV genotype prevalence varies in different periods, even within a geographic region. In Poland, the prevalence of subtype 1b decreased, while subtype 3a increased, over the past two decades [17]; in Germany, subtype 1b has gradually come to replace subtype 1a over the last 20 years since 1994 [18], while the prevalence of genotype 1 decreased during the periods 1996-2006 in Austria [19] and 1970-1990 in France [20]. Changes in human migration flow and transmission route are reportedly the major factors driving the variation in genotype prevalence [18, 20].

In China, the prevalence of anti-HCV (using a second-generation UBI enzyme-linked immunoassay) was estimated to be 3.2 % (~40 million) in the Chinese population [21]. In general, approximately 75 % of HCV-infected individuals go on to develop chronic hepatitis, of which 5-10 % eventually progress to cirrhosis or hepatocellular carcinoma [21, 22]. At present, 1b and 2a are the major HCV subtypes circulating in China, especially in the North and West [13, 23]. A population-based genetic estimate suggested that the subtype 1b major clusters A and B were introduced into China during the period 1966-1976 [24], followed by an increased prevalence of subtype 2a across the nation [25, 26]. The prevalence of subtype 2a decreased after 1994 when the IFN therapy for HCV treatment was implemented in China [27]. Subtype 6a was initially detected in Guangzhou in the Pearl River Delta region of South China in 2002 [13] and has become one of the dominant subtypes in this region since 2004 [28]. Given the economic importance of the Pearl River Delta region, located in Guangdong Province, it is speculated that the continuous socio-economic advancement and migration flow in this region may be influencing the genotype distribution pattern of HCV at both the local and national level.

Epidemiological monitoring of HCV genotype changes is critical to appropriately target disease control and prevention. Here, we report HCV genotype changes in China during two distinct time periods (2004-2007 and 2008-2011) and show that the subtype 6a frequency was significantly higher among blood donors in provinces other than Guangdong in the period 2008-2011 than it was in 2004-2007. This shift in HCV genotype indicates that more attention should be paid to subtype 6a, which has emerged in recent years in China, for the purpose of clinical drug and therapy research.

## Materials and methods

### Blood donors and samples

From December 2008 to August 2011, 770 serum samples that were HCV antibody positive by routine screening were collected from Chinese volunteer blood donors. HCV RNA was detected as described previously [29], which gave positive results for 501 donors [29]. This study has been approved by the Institutional Review Board at the Guangzhou Blood Center, and the guidelines set by this board were strictly followed. The physicians ensured that individuals were personally interviewed to assure their complete understanding of the informed consent, and the participants provided their written consent. The study protocol conformed to the ethical guidelines of the 1975 Declaration of Helsinki and was approved by the Medical Ethics Committee of the Guangzhou Blood Center. Four hundred twenty samples with available genotyping information were used in further analyses. These 420 donors were stratified into Guangdong and non-Guangdong groups according to their province of birth. The 2004-2007 cohort was described in our pervious study [28].

### HCV RNA preparation and RT-PCR amplification

Viral RNA was extracted from 150 µl of serum using a QIAamp Viral RNA Mini Kit (QIAGEN Inc, Valencia, CA, USA). Reverse transcription PCR (RT-PCR) was performed using a k1622 First Strand cDNA Synthesis Kit (Fermentas, CA, USA) according to the manufacturer’s protocol. The cDNA was amplified using a nested PCR with E1- and NS5B-specific primers as described previously [28].

### HCV genotyping and phylogenetic analysis

The amplified products were sequenced using an Applied Biosystems (ABI) PRISM Big Dye Terminator Cycle Sequencing Ready Reaction Kit, Version 3.1 (Applied Biosystems, Foster City, CA, USA). The resulting sequences were aligned using BioEdit 5.0.9 (http://www.mbio.ncsu.edu/BioEdit/bioedit.htlm). Phylogenetic analyses were performed in MEGA 5, as described previously [30]. The neighbor-joining approach (using the HKY+I+Γ6 substitution mode) with 500 iterations of bootstrap sampling was performed. HCV genotype reference sequences were retrieved from the HCV database (http://hcv.lanl.gov/content/sequence/HCV/ToolsOutline.html). The following sequences in GenBank were used as references in the phylogenetic analysis: M62321 (1a), M58335(1b), D14853 (1c), AB047639,D00944 (2a), D10988 (2b), D50409 (2c), D17763 (3a), D49374 (3b), D63821 (3k), Y12083 (6a), D84262 (6b), EF424629 (6c), D84263 (6d), DQ314805 (6e), DQ278893 (6k), DQ278894 (6n), EF108306(7a).

### Statistical analysis

Statistical calculations were performed using SPSS for Windows, version 16.0 (SPSS, Chicago, IL, USA). The chi-square test was used to compare the HCV genotype differences between the 2004-2007 and 2008-2011 cohorts. Fisher’s exact test was used when the chi-square test condition was not satisfied. The mean age between the two cohorts was examined using Student’s t-test. *P*<0.05 was considered to be statistically significant in all tests.

## Results

### Characteristics of the 2008-2011 cohort population

**Fig. 1:**
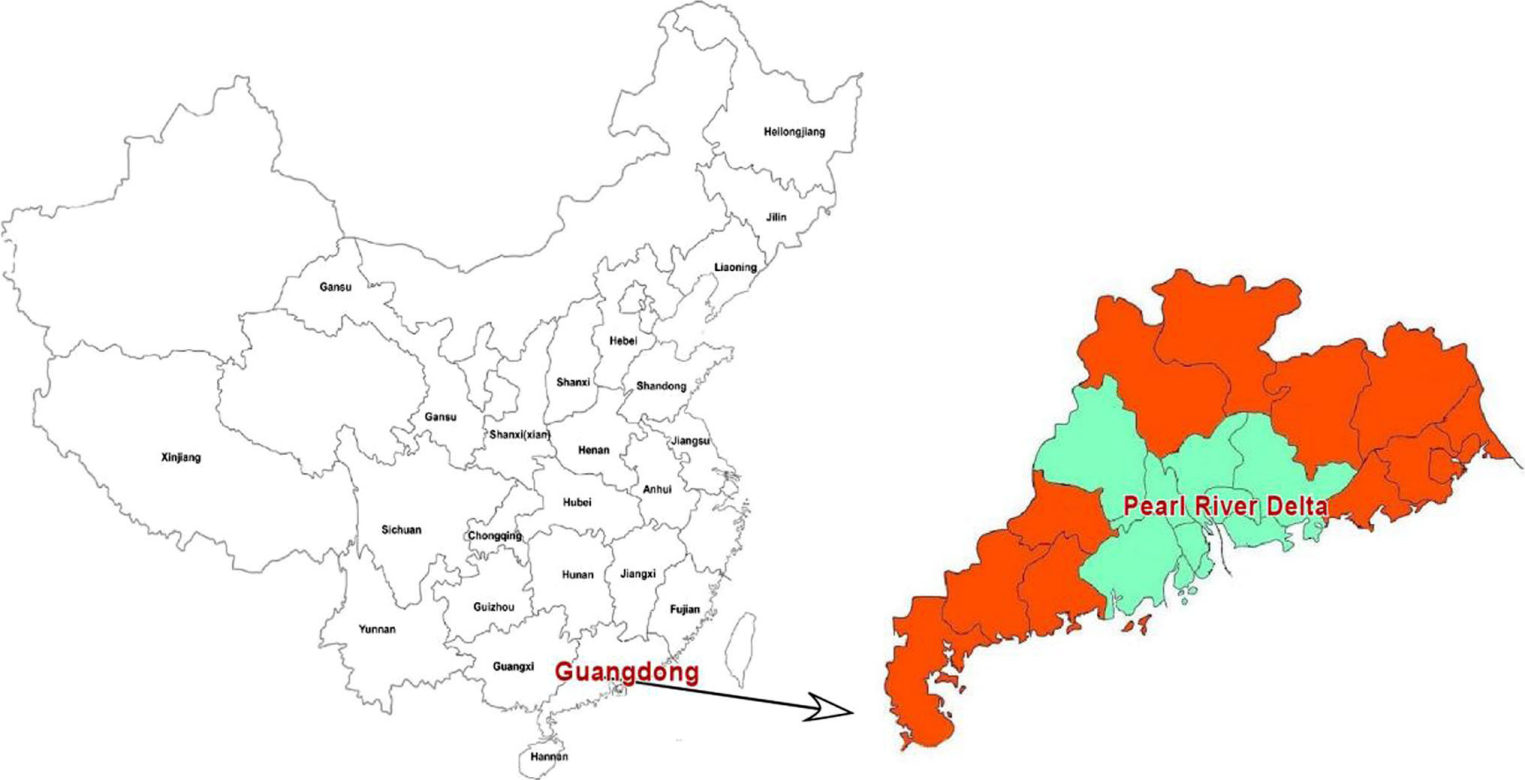
The geographic location of Guangdong Province and surrounding regions in China. The highlighted provinces shown in the diagram were included in the present study

The geographic distribution of Guangdong Province and the other study regions in China are shown in Fig. 1. The age, gender and geographical information are summarized in Table 1. Of the 420 HCV-infected blood donors, 80.5 % (338/420) were male and 19.5 % (82/420) were female, while 61.4 % (258/420) were from Guangdong and 38.6 % (162/420) from other regions. Based on the policy for voluntary blood donation in China, donors aged 18-55 years were recruited. The mean age of the donors was 32.7 ± 8.9 years.

**Table 1:** HCV genotype information for the 2008-2011 cohort

Genotype	Subtype (n)	Region		Gender		Age	
		Guangdong n (%)	Non-Guangdong n (%)	Male n (%)	Female n (%)	<40 years n (%)	≥40 years n (%)
Genotype 1	1a (2)	1 (0.4)	1 (0.6)	144 (77.4)	42 (22.6)	133 (71.5)	53 (28.5)
	1b (184)	113 (43.8)	71 (43.8)				
Genotype 2	2a (32)	7 (2.7)	25 (15.4)	22 (68.8)	10 (31.2)	19 (59.4)	13 (40.6)
Genotype 3	3a (35)	26 (10.1)	9 (5.6)	44 (78.6)	12 (21.4)	46 (82.1)	10 (17.9)
	3b (21)	9 (3.5)	12 (7.4)				
Genotype 6	6a (144)	101 (39.1)	43 (26.5)	128 (87.7)	18 (12.3)	114 (78.1)	32 (21.9)
	6n (1)	0 (0)	1 (0.6)				
	6e (1)	1 (0.4)	0 (0)				
Total	420	258 (100)	162 (100)	338 (80.5)	82 (19.5)	312 (74.3)	108 (25.7)
χ^2^, *P*-value		33.570, 2.97E-6*		8.846, 0.031		7.389, 0.06	

The results are stratified by gender, age and geographic region

* Data were obtained by Fisher’s exact test

### Genotyping information of the 2008-2011 cohort

Both E1 and NS5B sequences were obtained from 403 samples. For three samples, only the E1 sequence was obtained, and for 14 samples, only the NS5B sequence was obtained. The genotypes of these sequences included 1a, 1b, 2a, 3a, 3b, 6a, 6n and 6e (Supplemental Figure 1a and b), from which partial NS5B and E1 HCV sequences were obtained previously [31]. Of these sequences, 1b and 6a were the major subtypes (43.8 % and 34.3 %, respectively), followed by 3a (8.3 %), 2a (7.6 %), 3b (5 %), 1a (0.5 %), 6n (0.3 %) and 6e (0.2 %). No co-infection or recombination between genotypes or subtypes was found in this study (Table 1).

We observed a significant difference in subtype prevalence between Guangdong and non-Guangdong donors (χ^2^=33.570, *P*= 2.97E−6) in 2008-2011. Subtype 6a was more common among the Guangdong donors (χ^2^=7.235, *P*=0.007), while subtype 2a was more common in the non-Guangdong donors (χ^2^=22.872, *P*=1.73E−06). The male/female ratio was higher in genotype 6 than in the genotype 2 (χ^2^=7.089, *P*=0.008) or genotype 1 group (χ^2^=5.806, *P*=0.016). There was no significant difference in genotype distribution between the younger (<40 years) and older donor groups (≥40 years) (χ^2^=7.389, *P*=0.06).

### Comparison of the genotype distribution pattern between the 2004-2007 and 2008-2011 cohorts

**Fig. 2:**
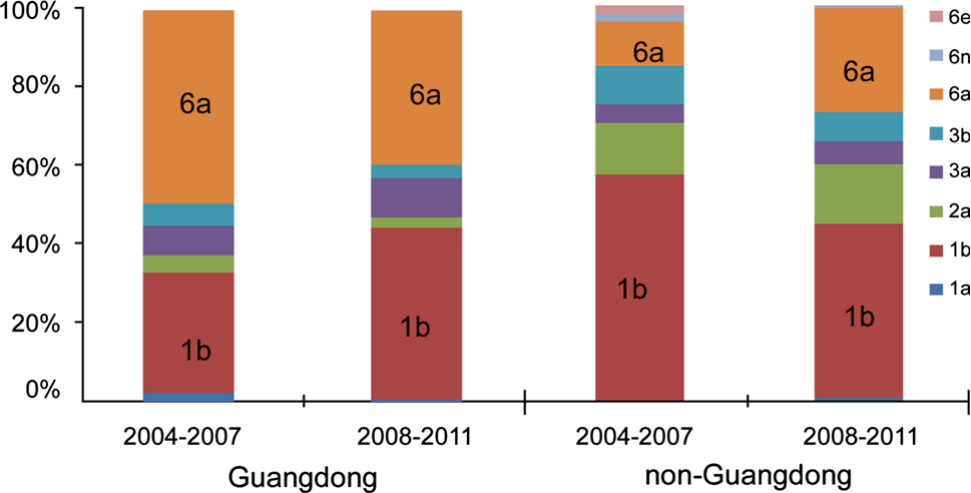
HCV genotype distribution in the Guangdong and non-Guangdong donors between the 2004-2007 and 2008-2011 cohorts. Subtype 6a was more common in the non-Guangdong region in the period 2008-2011 than 2004-2007. Subtype 1b exhibited a marginal decrease in the non-Guangdong region during 2008-2011

In order to examine the potential genotype shift in HCV that might have taken place in China in recent years, the 2008-2011 cohort was compared to the previously reported 2004-2007 cohort [28]. In the latter case, subtypes 1a, 1b, 2a, 3a, 3b and 6a among the Guangdong donors accounted for 2.1 %, 31.0 %, 4.1 %, 7.6 %, 5.5 % and 49.7 % of all of the subtypes, respectively. Among the non-Guangdong donors, subtype 1b, 2a, 3a, 3b, 6a, 6n and 6e accounted for 57.1 %, 13.2 %, 4.4 %, 9.9 %, 11.0 %, 2.2 % and 2.2 %, respectively [28]. Between the 2004-2007 and 2008-2011 cohorts, there was no significant difference in subtype distribution among the Guangdong donors (χ^2^=11.569, *P*=0.058), while the non-Guangdong donors did show significant differences (χ^2^=14.990, *P*=0.023). In particular, subtype 6a became significantly more prevalent in the 2008-2011 cohort of non-Guangdong donors (χ^2^=8.513, *P*=0.004) (Fig. 2).

**Fig. 3:**
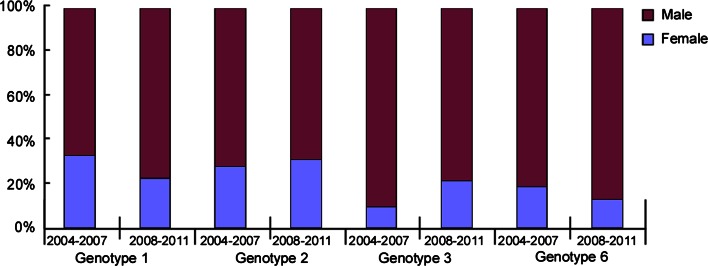
Gender distribution of genotypes 1, 2, 3 and 6 in the 2004-2007 and 2008-2011 cohorts. No significant difference was found in the groups stratified by gender (male vs. female) for genotypes 1, 2, 3 and 6 between the two cohorts

**Fig. 4:**
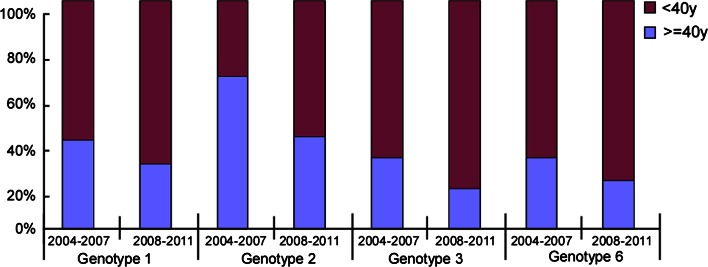
Age distribution of genotypes 1, 2, 3 and 6 between the 2004-2007 and 2008-2011 cohorts. No significant difference was found in the groups stratified by age (<40 years vs. ≥40 years) for genotypes 1, 2, 3 and 6 between the two cohorts

The mean age of the 2008-2011 cohort was lower than that of the 2004-2007 cohort (32.7 ± 8.9 years vs. 34.4 ± 6.8 years [28], *P*<0.01). No significant difference was found in the groups stratified by gender (male vs. female) or age (<40 years vs. ≥40 years) for genotype 1, 2, 3 and 6 between the two cohorts (Figs. 3 and 4).

## Discussion

Approximately 3.2 % (40 million) of the Chinese population are HCV infected [21]. This number has continued to increase in recent years due to migration and changes in the routes of transmission. With no effective vaccine or post-exposure prophylaxis for HCV treatment, limiting transmission is a primary strategy for the prevention and control of HCV epidemics. Importantly, epidemiological surveillance of HCV transmission routes is instrumental in tracking the dynamics of viral changes in various epidemiological settings [32–34]. In this study, we report the genotype differences between two time periods, 2004-2007 and 2008-2011, in one of China’s HCV epicenters, Guangdong Province.

Guangdong Province is located in the southeast of China, neighboring the Guangxi, Hong Kong, Macau and Fujian regions (Fig. 1). This was the first region in China to undergo economic reform in 1978, resulting in profound social change and an influx of millions of immigrant workers [35]. These changes undoubtedly influenced the epidemiology of infectious diseases, both locally, and in China as a whole. While the “imported” subtype 6a was initially detected in Guangdong Province, the distribution pattern of the HCV genotype has changed in China. In this study, we found that in the non-Guangdong regions, HCV subtype 1b was less commonly found during 2008-2011 than in the 2004-2007 period (χ^2^=4.136, *P*=0.042). But this difference was not significant after Bonferroni correction, with *P* not less than 0.01. In line with our results, the prevalence of subtype 1b was also found to have decreased in two cities of central China: Chongqing, during 1992-2011, and Xi’an, during 2000-2009 [36, 37]. The trend of a decreasing subtype 1b was also reportedly observed over the last 5-10 years in other Asian countries, as well as in Australia and Egypt [19].

The factors driving the decreasing prevalence of subtype 1b in Guangdong Province are not clear. One plausible explanation is a change in the transmission route from blood transfusion and/or blood products, operations, surgeries and dental procedures to a route of intravenous drug use (IVDU) and high-risk sexual behavior (HRSB) [18, 20]. Since 1998, improved control of blood transfusion in China has decreased HCV transmission by the general route that was mostly associated with subtype 1b over the other subtypes. Furthermore, the decreased prevalence of subtype 1b may be beneficial in terms of treatment outcome. Genotype 1 has been shown to be more often refractory to treatment than genotypes 2 and 3. Genotype 1, especially subtype 1b, was shown to have only a 55 % average response rate to IFN and RBV therapy, while this rate was much higher (70 %) in cases of infection with genotypes 2 and 3 [14, 15].

The present study also identified an increasing prevalence of subtype 6a in donors originating from provinces other than Guangdong, suggesting an outward flux of 6a from Guangdong to other regions of China. This is one of the few studies that have examined the distribution change of subtype 6a in China or Southeast Asia. Subtype 6a in China was estimated to have been transmitted via the IVDU route from Vietnam to the Chinese provinces of Guangxi and then to Guangdong [35]. Our previous study also showed the subtype-6a-infected population expanded from IVDU to the general population, and subtype 6a is largely responsible for the regional epidemic in Guangdong Province [30]. In the 2008-2011 cohort reported here, we found that the prevalence of subtype 6a was increasing in the non-Guangdong regions, indicating that 6a has spread from Guangdong to other regions. One piece of supporting evidence is that genotype 6 was previously found to have spread via the IVDU route to Hong Kong, a region in close proximity to Guangdong [33, 34]. Factors driving the increasing prevalence of subtype 6a in the non-Guangdong region may include socio-economic development and migration between Guangdong and other regions in China. Additionally, the higher viral load found in subtype-6a-infected patients compared to genotype-2- or 3-infected patients may also contribute to a higher transmission rate of subtype 6a [31, 38]. Hence, once subtype 6a was established in the non-Guangdong regions, it may have gradually overtaken the other subtypes. This study demonstrates an increasing prevalence of subtype 6a in the non-Guangdong region and underscores the importance of continuous surveillance of subtype 6a for the purpose of designing genotype-specific therapies. Previously, several studies reported that treatment using IFN and RBV therapy for 24 or 48 weeks appeared to have a similar response rate in patients infected with genotypes 2, 3 and 6, but it was higher in the case of genotype 1 patients [39–42]. However, in European and North American clinical practice, genotype 6 infections are treated according to protocols used for genotype 1 because of the lack of treatment efficacy data on genotype 6 [43].

In the present study, we also observed that the median age of the 2008-2011 cohort was younger than that in 2004-2007. This might be caused by the change in the HCV transmission route from blood transfusion to IVDU and sexual transmission (especially from men to men, MSM) in young people in Europe, the United States, Australia and Southern China over the past 5-10 years [44–46]. Our study highlights the critical importance of effective HCV prevention and control strategies among young people, in particular focusing on the IVDU and MSM transmission routes.

In conclusion, a significant temporal change in the prevalence of HCV genotypes over time has recently taken place in China. This study provides important information for the development of improved HCV prevention and control strategies.

## Electronic supplementary material

Below is the link to the electronic supplementary material.

Supplementary material 1 (FAS 240 kb)

Supplementary material 2 (FAS 161 kb)

Supplemental Figure 1 Phylogenetic tree constructed from (1a) E1 and (1b) NS5B nucleotide sequences. Bootstrap values are shown in the tree root. The scale bar represents 0.05 nucleotide substitutions per site. GZ- means the strains from Guangdong, n-GZ- means the strains from non-Guangdong. (Reference strains are represented in red circles) (PDF 59 kb)
