# Supplementary figures and images for: Increased prevalence of hepatitis C virus subtype 6a in China: a comparison between 2004–2007 and 2008–2011
Source: Arch Virol. 2014 Aug 2;159(12):3231–7. doi: 10.1007/s00705-014-2185-1 (PMC4221604; doi:10.1007/s00705-014-2185-1)

Supplemental Figure 1

1a

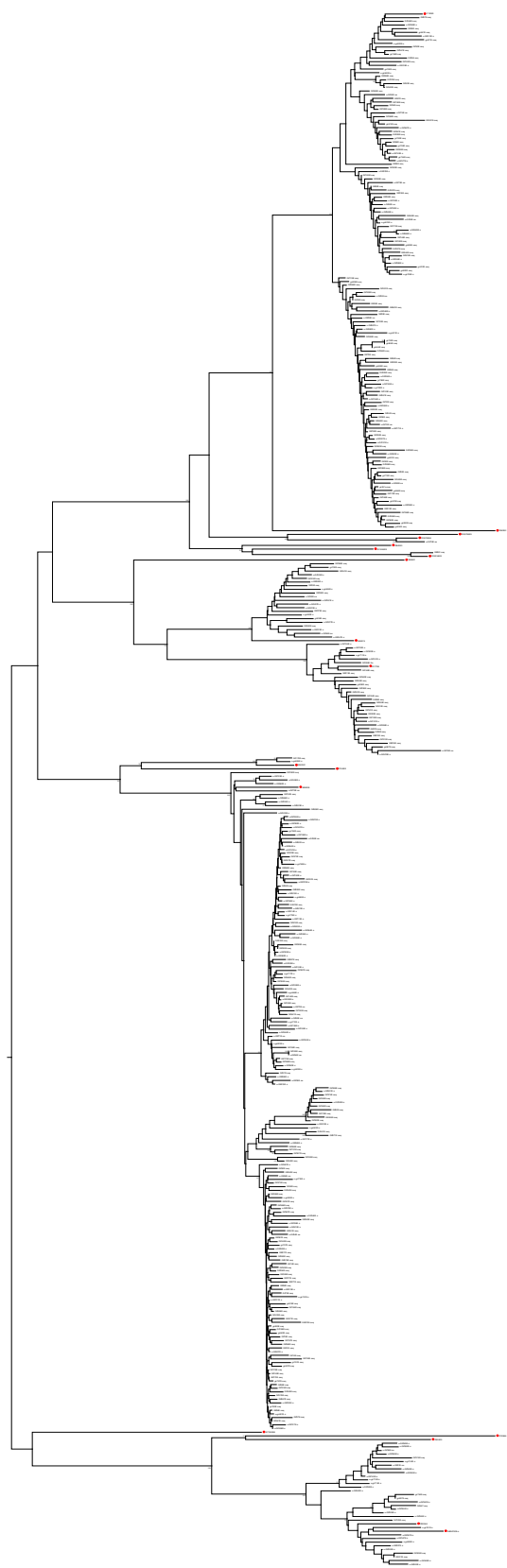

1b

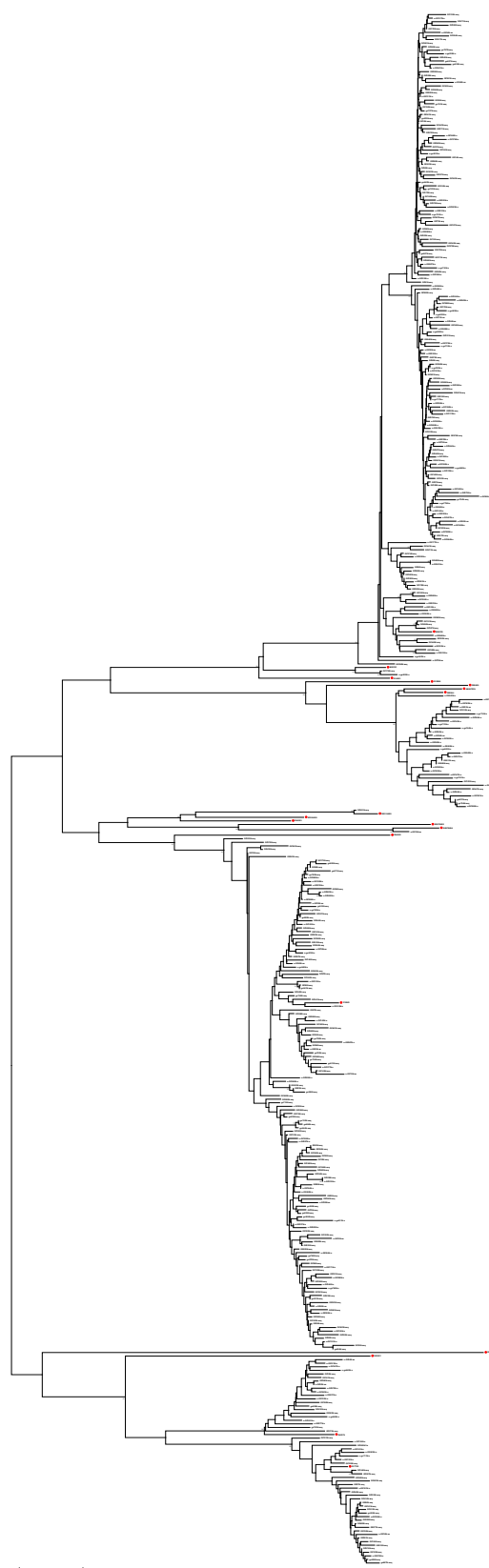

Supplement: Supplementary file 3 — Supplemental Figure 1 Phylogenetic tree constructed from (1a) E1 and (1b) NS5B nucleotide sequences. Bootstrap values are shown in the tree root. The scale bar represents 0.05 nucleotide substitutions per site. GZ- means the strains from Guangdong, n-GZ- means the strains from non-Guangdong. (Reference strains are represented in red circles) (PDF 59 kb) [file 705_2014_2185_MOESM3_ESM.pdf]
